# Psychometric Properties of the MSLQ-B for Adult Distance Education in China

**DOI:** 10.3389/fpsyg.2021.620564

**Published:** 2021-02-23

**Authors:** Ying Zhou, Jianhua Wang

**Affiliations:** ^1^School of Education, Beijing Open University, Beijing, China; ^2^School of Foreign Languages, Renmin University of China, Beijing, China

**Keywords:** learning strategies, questionnaire, validation, adult distance learning, psychometrics

## Abstract

In the education context, The Motivational Strategies for Learning Questionnaire (MSLQ) is extensively used in assessing self-regulated learning strategies. However, more research is needed to address whether it is applicable for distance education. The exploratory and confirmatory factor analysis were used to test the Chinese version of the Motivated Strategies for Learning Questionnaire part-B for distance learning (MSLQ-B-DL) using two samples totalling 385 participants. This paper substantiates MSLQ-B-DL's criterion-related, convergent, and factorial validity, as well as its internal consistency, in China. Specifically, the concurrent validity of the MSLQ-B-DL was shown from three aspects: (a) the negative correlation of MSLQ-B-DL with trait procrastination; (b) the positive correlation of MSLQ-B-DL with self-control; and (c) the positive correlation of MSLQ-B-DL with instrumental help-seeking and the former's negative correlation with help-seeking avoidance and executive help-seeking. Finally, this study highlights the MSLQ-B-DL's validity and reliability in evaluating the learning strategies in adult distance education in China.

## Introduction

Distance learning is the education of students who may not always be physically present at a school. Distinct from face-to-face education, distance learning requires students to adopt self-regulated learning (SRL) behaviors (Adam et al., 2017). As a result, distance learning is considered to improve learners' motivation and their academic performance (Hartnett, 2016). Over the past 30 years, research has proposed various theoretical models to explain the process of self-regulated learning. Until recently, there were six theoretical models describing self-regulated learning: models from Zimmerman (2000), Boekaerts (2011), Winne and Hadwin (2008), Pintrich (2000), Efklides (2011), and Hadwin et al. (2018).

The researcher Panadero (2017) conducted a comparative study of the six models listed above and found that the models from Pintrich (2000) and Zimmerman (2000) are the most popular. They are similar in terms of the elements of self-regulation, and the role of context. However, Pintrich's model is more comprehensive, easier to understand, and more applicable to the classroom setting (Dignath and Büttner, 2008) than Zimmerman's model. It contains a complete description of different types of sub-processes and proposes more specific sub-processes, including motivation and strategies. Pintrich's model emphasizes the difference between each stage and its sub-processes, believing that each stage has unique characteristics. Besides, it treats motivational process an independent variable which interacted with cognitive, behavioral, and contextual factors to affect self-learning (Pintrich et al., 1993a). According to this model, more specific intervention measures can be proposed. Moreover, it focuses more on metacognition and is utilized in investigating the target orientation's function in SRL (Pintrich and de Groot, 1990). For these reasons, this study is based on Pintrich's model. For these reasons, this study is based on Pintrich's model in order to facilitate research comparison and theory correction.

According to theoretical and empirical research, learning strategies are important predictors of learning performance in the online environment. Learning strategies are processes that involve generating, organizing, or converting data for academic achievement (Neroni et al., 2019). SRL strategies refer to the application of resource management and cognitive strategies to supervise learning (Panadero, 2017). Research shows that there is a positive relationship between self-regulated learning strategies in the distance learning environment and academic performance (Broadbent and Poon, 2015; Broadbent, 2017). Studies on distance learning tend to focus on domain-general strategies and have found that students use these self-regulated strategies to benefit their academic achievement (e.g., Lin et al., 2017). However, students practice distinct forms of learning strategies to fulfill various academic goals (Neroni et al., 2019). In the context of academic performance, extensive research has explored the most effective as well as the unfavorable learning strategies (e.g., Matcha et al., 2019). Nevertheless, the majority of these studies were performed on undergraduate students on campus, and few explore the issue in the context of distance education.

Determining which learning strategies deliver the most optimal benefits for academic performance is vital for students and their teachers who can provide valuable support accordingly (Donker et al., 2013). Different classifications of learning strategies have been developed over time. For example, learning strategies can be classified into resource management, metacognitive, and cognitive strategies (McKeachie et al., 1987).

The Motivational Strategies for Learning Questionnaire (MSLQ) is a widely used tool to measure learning strategies used by students because of its good psychometric properties for different samples (Jackson, 2018; Soemantri et al., 2018). There are two versions of the MSLQ that have been widely translated into different languages and used in research: one version is for college students and consists of 15 subscales and 81 items (Pintrich et al., 1993b), and the other version is for junior high students and consists of five subscales and 44 items (Pintrich and de Groot, 1990). For young students, both the full version and specific subscales are applicable. For example, Pintrich and de Groot (1990) examined the motivational factors (internal value, self-efficacy, and test anxiety) and self-regulated learning factors (strategy use and self-regulation) in a sample of young students. The MSLQ has been translated and applied in many countries, such as Australia (Martin and Marsh, 2006), China (Yang et al., 2012; Tong et al., 2019), and Egypt (Shawer, 2013).

Through the use of the MSLQ in many studies, two concerns have been raised. First, social cognitive theory proposes that students' motivation and learning strategies are context-specific. However, the MSLQ is designed for all subjects and all kinds of learning activities, failing to account for context. Second, psychometrics problems have arisen in some studies, where it has been found, for example, that the partial molecular scale of the MSLQ lacks discriminant validity and that the factor structure of latent variables is not stable (Hilpert et al., 2013). In addition, in a study using the MSLQ in a sample of 611 college students, exploratory factor analysis showed that for Chinese adult learners, the factor of internal goal orientation disappears, the factor of peer learning is not very stable, and the factor of time and environment management is actually two factors (Tong et al., 2019).

There are relatively few empirical studies on distance learning strategies. Each scale has different measurement standards, Kizilcec et al. (2016) believe that it is not practical to create a new questionnaire to measure existing concepts or to use a complete but lengthy scale in an attempt to obtain high quality answers. Moreover, each study is based on a unified understanding of the construct and structure of learning strategies, without considering the difference between traditional learners and distance learners.

Recently, researchers have used the MSLQ to measure students' self-regulated learning in an online environment. Based on these studies, one option is that distance education studies could use either the MSLQ's subscale of learning strategies or a combination of items from the MSLQ. Cho and Summers (2012) used the complete version of the MSLQ to study learning strategies in distance education, but found that the structure did not fit the sample well. Because the MSLQ was originally developed for traditional face-to-face education, it is possible that many items do not directly reflect the learning behaviors of distance learners. Recognizing this possibility, Meijs et al. (2019) revised the subscales of the MSLQ to conform to distance education. Factor analysis of the new learning strategy questionnaire for distance education (MSLQ-B-DL) revealed five factors. Further research (Neroni et al., 2019) proved the stability, reliability, and validity of its factor structure.

In line with this context, this paper aims to ensure the validity and reliability of MSLQ-B-DL's Chinese version (Meijs et al., 2019). Specifically, this study will investigate factor validity, convergence validity, internal consistency, and criterion-related validity of the MSLQ-B-DL. For the reasons stated below, convergence validity will be investigated by calculating the correlation between distance learning strategies and procrastination, self-control, and help-seeking behaviors; criterion-related validity will be examined by calculating the correlation between distance learning strategies and academic performance.

### Construct Validity

As for construct validity, this study examines three constructs that are related to distance learning strategies: procrastination, self-control, and help-seeking behavior.

#### Procrastination

Achievement goal theory suggests that one result of adopting goal orientation is the differential use of learning strategies (Pintrich et al., 2000). Therefore, learning strategies are related to self-regulated learning and procrastination. Since the use of learning strategies consumes energy and time, students with little motivation for distant tasks may not apply learning strategies frequently. Schouwenburg (2004) proposed that procrastination is negatively correlated with the systematic and disciplined approach to word as well as the planning and management of time, which were all manifestations of poor organization. As a part of conscientiousness in an individual's personality, organization is negatively correlated with procrastination (Steel, 2007). Procrastination is linked to the reduced application of metacognitive and cognitive strategies (Limone et al., 2020). Therefore, this study hypothesizes that distance learning strategies are negatively correlated with procrastination.

#### Self-Control

Self-control is related to a broad spectrum of behaviors. Empirical studies have demonstrated that people with high self-control are more skilled in emotion regulation as well as thought and impulse control than their low self-control counterparts (Robson et al., 2020).

An essential mediating variable between perceived ability and learning engagement could be the perceived academic control, which describes a person's perception of his or her capacity to impact and forecast academic performance (Respondek et al., 2020). In the former perspective, ability can be improved by learning and effort, which promotes a higher sense of academic control. Therefore, this study hypothesizes that distance learning strategies are positively correlated with self-control.

#### Help-Seeking Behavior

Help-seeking behavior, as opposed to dependence, is regarded as an important self-regulated learning strategy (Karabenick and Gonida, 2018). This recognition began by distinguishing instrumental help-seeking behavior (Sideridis and Stamovlasis, 2016), which involved help-seekers' requests for others to explain problem-solving method. By contrast, learners with executive help-seeking requested for others to solve problem for them. And learners with avoidance of help-seeking did not seek help. Thus, in the context of distance learning strategies, this study theorizes their (a) negative correlation with help-seeking avoidance and executive help-seeking, and (b) positive correlation with instrumental help-seeking.

### Criterion-Related Validity

For criterion-related validity, this study adopted academic performance in the course to examine the predictive criterion-related validity of the scale. A study that used the MSLQ with Chinese adult learners suggested that future studies use academic performance to verify the predictive validity of the MSLQ (Tong et al., 2019). In distance learning, time management learning strategies, metacognitive strategies, and effort regulation strategies can improve academic performance (Broadbent and Poon, 2015). These approaches assist learners in processing and storing information systematically (Dignath and Büttner, 2008). The usage of the learning strategies aims at improving academic performance. Therefore, this study hypothesizes that distance learning strategies are positively related to academic performance.

## Methods

### Translation

Two graduate students majored in English translated the original MSLQ-B-DL (Meijs et al., 2019) into Chinese independently. The “translate and back translate” procedure was used to get a readable Chinese expression of the same meaning.

### Participants and Procedures

There were 150 participants (113 women) in sample A, who were recruited from an adult distance learning course entitled “Psychology.” It's a self-paced course of the undergraduate degree program from an open university in China. All participants provided informed consent and were financially compensated for their participation. All participants took the course in the autumn of 2018, filled out e-questionnaires at the beginning of the semester. One hundred and fifty eight people participated in the survey, 150 of whom were valid, with a 95% response rate. The academic ethics committee of the first author's institution reviewed and approved this study. The data from sample A were used in the exploratory factor analysis (EFA).

There were 236 participants (143 women) in sample B. All participants studied the course in the spring semester of 2019, filled in questionnaires at the beginning of the semester, and took part in the course examination at the end. Two hundred and forty one people filled in the questionnaire, 5 of whom were absent from the exam, with a response rate of 98%. The data from sample B were used in the confirmatory factor analysis (CFA) and validity test. The participants' characteristics for both samples are presented in Table 1.

**Table 1:** Demographics of two samples by gender, age, and experience with distance learning.

		**Sample A**	**Sample B**
Gender	Male	37	93
	Female	113	143
Age	18–25 yrs.	28	32
	26–30 yrs.	27	51
	31–40 yrs.	78	122
	41–50 yrs.	17	30
Online learning experience	<1 year	56	83
	1–2 years	69	106
	3–4 years	14	38
	>4 years	11	8

Concerning the sample size, the N:p ratio is used in EFA, i.e., of participants (N) to variables (p) set traditionally to 5:1 (Kyriazos, 2018). A CFA rule of thumb is the ratio of cases to free parameters, or N:q is commonly used for minimum recommendations and 10:1–20:1 is a commonly suggested ratio (Schumacker and Lomax, 2015).

### Measures

#### MSLQ-B-DL

The MSLQ-B-DL (Meijs et al., 2019), which was adapted from the MSLQ, measures the learning strategies of distance learners through 25 items. A 6-point Likert scale was used in both samples (1 = *strongly disagree*, 6 = *strongly agree*), with higher scores representing better use of learning strategies. The MSLQ-B-DL had acceptable reliability in this sample (see the “Result” section). Both English and Chinese versions of the scale are presented in Appendix 1.

#### General Procrastination Scale (GPS)

The General Procrastination Scale (Sirois et al., 2019) measures trait procrastination and contains 9 items. A 6-point Likert scale was used in both samples (1 = *strongly disagree*, 6 = *strongly agree*), with higher scores representing higher levels of procrastination tendency. The GPS had excellent reliability with Cronbach's α as 0.83 in sample B.

#### Self-Control

The self-control scale (Tan and Guo, 2008) measures the self-control ability of university students and consists of 19 items. A 6-point Likert scale was used in both samples (1 = *strongly disagree*, 6 = *strongly agree*), with higher scores representing higher levels of self-control ability. This scale was reliable with Cronbach's α as 90 in sample B.

#### Help-Seeking Behavior

The help-seeking behavior scale (Tang, 2004) contains 14 items, and includes three dimensions: instrumental help-seeking, executive help-seeking, and help-seeking avoidance. A 6-point Likert scale was used in both samples (1 = *strongly disagree*, 6 = *strongly agree*). Higher scores for instrumental help-seeking represent more positive aspects of help-seeking behavior. Higher scores for executive help-seeking and help-seeking avoidance represent more negative aspects of help-seeking behavior. The math learning terms from the original scale were replaced with “online learning.” For example, “for me, online learning will be easier with the help of others.” The Cronbach's α with the sample B was 0.82.

#### Academic Performance

Fifty questions were selected from the course database to form the knowledge test, ensuring that questions were drawn from all of the course units. All questions were single choice questions. If the answer was correct, the participant earned 2 points. The scores ranged from 36 to 98, with an average score of 76.91 (*SD* = 11.64).

#### Demographic Variables

Demographic variables included gender, age, and online learning experience.

#### Data Analysis

Explorative factor analysis, descriptive analysis and correlations were used with SPSS 21.0. Confirmative factor analysis was used with AMOS 24.0. McDonald ω and Cronbach's α was calculated with Jamovi 1.6.9.

## Results

### Exploratory Factor Analysis (EFA)

First, factor validity was analyzed using sample A (*N* = 150), and the critical ratio (CR) and discrimination index (D) were used to analyse 25 items. The *t*-tests of the 25 items were all significant, and the correlations of the 25 items ranged from 0.67 to 0.89.

Construct validity was evaluated using the EFA, also with sample A. This paper adopted Bartlett's test of sphericity and the Kaiser-Meyer-Olkin (KMO) index to assess the sampling adequacy. The sample was found adequate based on the KMO value at 0.87, which was > 0.5 (Kaiser, 1974), and the statistically significant Bartlett's test of sphericity value [χ^2^(190) = 2106.35, *p* < 0.001]. Through a screen plot and the Promax rotation, we extracted the EFA's latent factors by maximum likelihood. As shown in Table 2, there were five factors with eigenvalues above 1 (range from 1.03 to 8.31), and these five factors explained 73.37% of the total variance of the 25 items. The McDonald's ω is 0.85. The composite Cronbach's α is 0.93. The average variance extracted (AVE) range from 0.45 to 0.66. The composite reliability (CR) ranges from 0.68 to 0.94. Only the Time management factor's AVE value is 0.45 which is < 0.50. And its composite reliability (CR) value is 0.71. If AVE is <0.5, but composite reliability is higher than 0.6, the convergent validity of the construct is still adequate (Fornell and Larcker, 1981).

**Table 2:** Standardized factor loading of exploratory factor analysis and internal consistency with Sample A.

**Items**	**Factor 1**	**Factor 2**	**Factor 3**	**Factor 4**	**Factor 5**
63	0.79				
72	0.78				
61	0.77				
67	0.74				
64	0.71				
59	0.69				
81	0.67				
76	0.64				
75		0.89			
58		0.82			
45		0.81			
50		0.79			
74			0.91		
73			0.88		
70			0.86		
36				0.77	
32				0.69	
38				0.66	
60					0.86
80					0.81
Eigen value	8.31	2.17	1.87	1.30	1.03
Explained variance	41.55%	10.83%	9.32%	6.53%	5.13%
Internal consistency	0.91	0.90	0.94	0.71	0.64

Five items (items 71, 43, 46, 47, and 51) were deleted from the eventual final questionnaire because the loadings were lower than 0.4, or cross-loading on more than one factor and the loadings were above 0.3. The loadings of the remaining 20 items were all above 0.50, among which the loadings of 17 items were above 0.70.

### Confirmatory Factor Analysis (CFA)

The CFA was conducted on sample B, using AMOS 24.0. As shown in Table 3, model fit was measured using the Satorra-Bentler scaled chi-square (χ^2^ = 370.315), comparative fit index (CFI = 0.93), Tucker-Lewis index (TLI = 0.92), and root mean square error of approximation (RMSEA = 0.07). The goodness-of-fit indices of the CFA model were acceptable (Baloglu, 2016).

**Table 3:** Fit Indices for the CFA of the MSLQ-B-DL.

	**χ^2^**	**d*f***	**χ^2^/df**	**CFI**	**NNFI**	**IFI**	**TLI**	**RMSEA**
MSLQ-B-DL(*N* = 235)	370.32^***^	160	2.31	0.93	0.92	0.93	0.92	0.07

*** *p < 0.001*.

**Figure 1:**
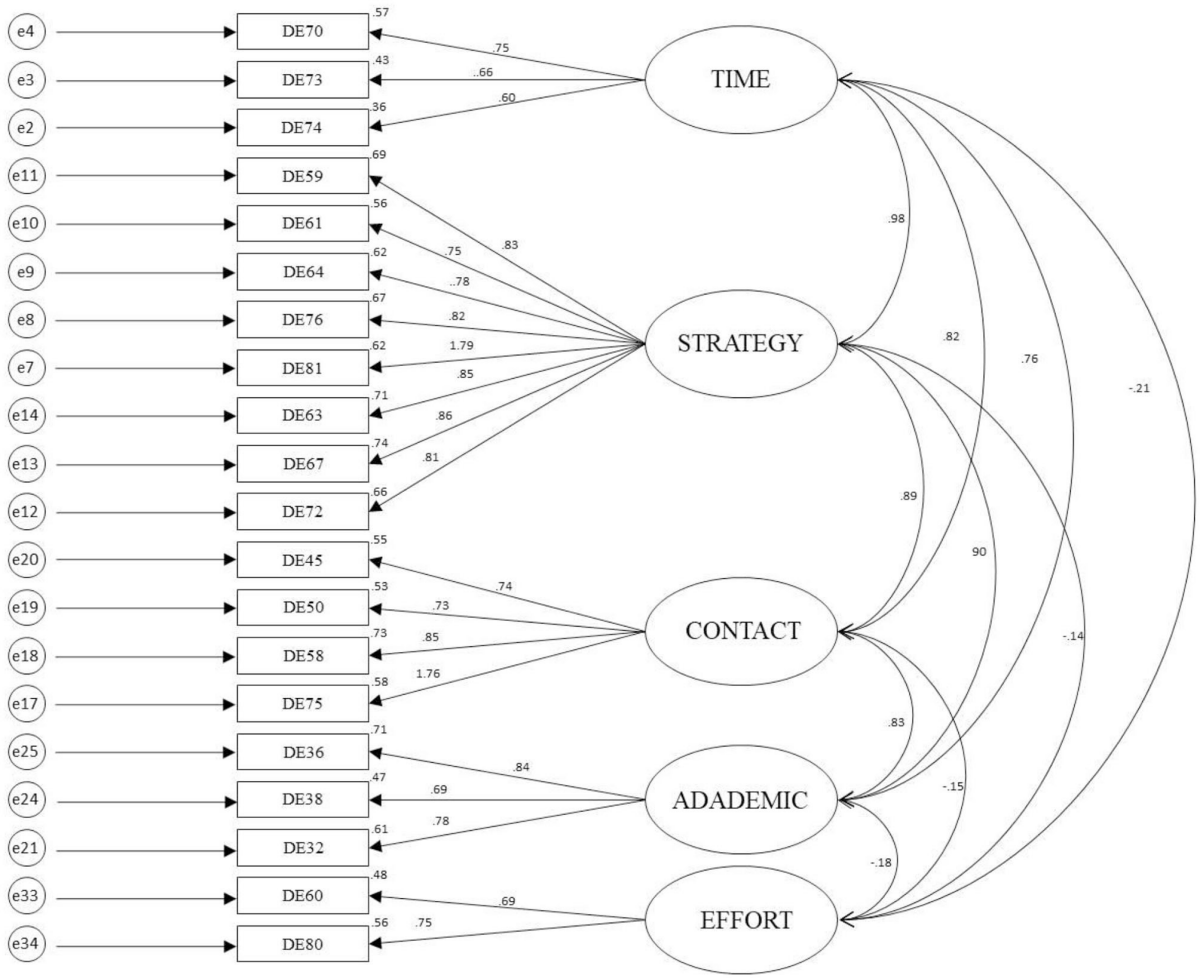
CFA for the MSQL-B-DL with standardized loadings.

Based on the items in the five factors, the factors were named as time management, effort regulation, cognitive strategy, critical thinking, and help seeking (see Figure 1).

The original time and environment management factor is divided into two factors: time management and effort regulation. The time management factor has three items. Item 74 (*Despite the dull and unexciting course materials, I can continue working until I complete the tasks*) belongs to effort regulation in the original questionnaire. However, in this study, participants paid attention to the deadline in the item. As a result, this item was considered to be related to time management factor. Distance learners may invest different resources and energies on time management and effort regulation than traditional students in a classroom setting. For example, adult online learners arrange their learning time in a highly autonomy. Besides, two of the reverse scoring items constitute the effort regulation factor.

The cognitive strategy factor constitutes of complex cognitive strategy and single cognitive strategy in the original MSLQ-B-DL (Davis et al., 2018). The eight items in subscale of the self-learning are also covered by the all the items of the complex and simple cognitive strategies in this study.

Critical thinking factor includes the original items, and another item (32: *As I revise the course readings, I organize my thoughts by outlining the material*) comes from the single cognitive strategy.

The fifth factor is help seeking. Four items in this factor were consistent with the original questionnaire. Help seeking is different from other learning strategies in that it is also social interaction. It can be defined as the process of seeking assistance from other individuals or other sources that facilitate accomplishing desired goals, which in academic context may consist of completing assignments or satisfactory test performance (Karabenick and Gonida, 2018).

### Internal Consistency and Convergent Validity

The Cronbach's alpha was calculated to evaluate the internal consistency of scores on the five factors. Cronbach's alpha estimates ranged from 0.64 to 0.94 for sample A, and from 0.71 to 0.94 for sample B.

Bivariate correlations between sample B's scores on the MSLQ-B-DL factors and scores on procrastination, self-control, and help-seeking behavior are displayed in Table 4. As hypothesized, correlations between the five factors of MSLQ-B-DL and procrastination were significant and negative. The correlations ranged from −0.18 to −0.49. Self-control was significantly correlated with cognitive strategy, help seeking, critical thinking, effort regulation, and the total score of MSLQ-B-DL. The relationships between instrumental help-seeking behavior and the five factors in MSLQ-B-DL were significant and positive. The analysis found (a) the significant and negative correlation of executive help-seeking behavior with effort regulation, and (b) the significant and negative correlation of help-seeking avoidance with effort regulation and the total MSLQ-B-DL score. These results provide strong convergent validity support for the MSLQ-B-DL.

**Table 4:** Correlation between MSLQ-B-DL, procrastination, self-control, and help-seeking behavior.

		**M**	**SD**	**1**	**2**	**3**	**4**	**5**	**6**	**7**	**8**	**9**	**10**
1	Time management	4.38	0.80	–									
2	Cognitive strategy	4.36	0.79	0.80^*^	–								
3	Help-seeking	4.37	0.79	0.65^*^	0.81^*^	–							
4	Critical thinking	4.21	0.88	0.57^**^	0.79^**^	0.70^**^	–						
5	Effort regulation	3.84	1.32	0.05	0.08	0.14^*^	0.00	–					
6	Total score of MSLQ-B-DL	4.24	0.65	0.77^**^	0.88^**^	0.84^**^	0.77^**^	0.47^**^	–				
7	Procrastination	2.80	0.85	−0.18^*^	−0.24^**^	−0.26^**^	−0.19^**^	−0.49^**^	−0.41^**^	–			
8	Self-control	4.24	0.84	0.11	0.18^**^	0.15^*^	0.19^**^	0.37^**^	0.31^**^	−0.68^**^	–		
9	Instrumental help-seeking	4.37	0.80	0.19^**^	0.22^**^	0.27^**^	0.15^*^	0.15^*^	0.27^**^	−0.39^**^	0.29^**^	–	
10	Executive help-seeking	3.55	0.79	−0.01	−0.05	−0.01	−0.06	−0.20^**^	−0.12	0.35^**^	−0.48^**^	0.28^**^	–
11	Help-seeking avoidance	2.67	1.03	−0.00	−0.02	−0.12	−0.02	−0.39^**^	−0.20^**^	0.54^**^	−0.75^**^	−0.26^**^	0.39^**^

* *p < 0.05*,

** *p < 0.01 (two-tailed)*.

### Criterion-Related Validity

Pearson correlations among gender, age, online learning experience, MSLQ-B-DL, and academic performance are shown in Table 5. Gender was negatively correlated with academic performance, indicating that male participants had better performance than female participants. Online learning experience was positively correlated with academic performance, indicating that academic performance increased with online learning experience. The total score of MSLQ-B-DL was positively related to academic performance, indicating that better use of online learning strategies improved performance.

**Table 5:** Pearson correlations between gender, age, experience, MSLQ-B-DL, and Academic Performance.

	**1**	**2**	**3**	**4**
1. Gender	–			
2. Age	0.14^*^	–		
3. Online learning experience	0.09	0.13^*^	–	
4. MSLQ-B-DL	−0.08	0.05	0.12	–
5. Academic performance	−0.15^*^	0.06	0.15^*^	0.88^**^

* *p < 0.05*,

** *p < 0.01 (two-tailed)*.

The results from regressing gender, age, online learning experience, and MSLQ-B-DL on academic performance are shown in Table 6. When gender, age, and online learning experience entered the model first, gender (β = −4.097, *p* < 0.01) and experience (β = 2.27, *p* < 0.05) were related to academic performance. When the total score of MSLQ-B-DL entered the model in the second step, gender remained significantly related to academic performance (β = −2.071, *p* < 0.01), and MSLQ-B-DL was significantly related to academic performance (β = 15.47, *p* < 0.001). In sum, the correlation and regression analyses provide support for the criterion validity of the MSLQ-B-DL.

**Table 6:** Hierarchical regression analyses with gender, age, and online learning experience entered at step 1, and total score of MSLQ-B-DL Entered at Step 2.

	**Step 1**		**Step 2**	
	β	t	β	t
Gender	−4.09	−2.65^**^	−2.07	−2.82^**^
Age	0.87	1.01	0.34	0.82
Online learning experience	2.27	2.41^*^	0.68	1.51
MSLQ-B-DL			15.47	28.22^***^
*R*^2^	0.05		0.79	
*ΔR*^2^			0.78	
*F*	4.28^**^		213.41^***^	

* *p < 0.05*,

** *p < 0.01*,

*** *p < 0.001 (two-tailed)*.

## Discussion

This study involves a total of 385 participants (129 men, and 256 women) in two different samples. This research demonstrates that the MSLQ-B-DL is a useful tool to measure online learning strategies of adult distance learners in China. The factorial validity of the MSLQ-B-DL was good. Moreover, the MSLQ-B-DL's factor structure, similar to its original version, was presented in the EFA results (Meijs et al., 2019). The results of the CFA demonstrate that the data fit well with the factor structure and the items' loadings are acceptable. The internal consistency indices of each factor in the two samples reach the appropriate standards, which are similar to the original MSLQ-B-DL. The MSLQ-B-DL has good convergence validity. The convergence validity criteria are procrastination, self-control, and help-seeking behavior. The total score of MSLQ-B-DL is negatively correlated with procrastination, executive help-seeking behavior, and help-seeking avoidance. Additionally, it is positively correlated with self-control and executive help-seeking behavior. This study also examined the correlation between adult distance learning strategy and academic performance. The Chinese version of the MSLQ-B-DL can distinguish between high and low performance, indicating that the questionnaire has acceptable predictive validity. The ability to measure differences is useful for research and practice, and for developing appropriate interventions for self-regulation.

To summarize, it is very necessary to emphasize students' self-regulated learning in distance education. It is of great significance to guide students to formulate personalized learning goals according to their own actual conditions. The previous MSLQ survey targets at traditional college students, which is not suitable for adult distance learners, who can determine their own time, place and learning pace. The newly developed questionnaire is based on a based on a mature questionnaire, and adapted to adult distance learners' online learning environment and learners' characteristics. The development of measurement tools in Chinese version not only contributes to the comparability of research results across cultures, but also has a guiding role for students who study independently on the Internet.

The Chinese version of the scale is different from the original scale in terms of two factors and items. Firstly, time management and effort management become two factors. The result is consistent with the Spain version (Ramírez-Dorantes et al., 2013) and Chinese version (Tong et al., 2019) of the MSLQ. Reverse scoring items constitutes the effort regulation factor, which is consistent with a previous study. Chinese subjects tend to be more careful when answering reverse scoring questions (Law et al., 2008). The self-regulated learning theory asserts that learners will invest their efforts flexibly to learn better (Panadero, 2017). The effort regulation strategy is one of the most important self-regulated learning strategies for online learners (Kizilcec et al., 2016). In addition, an earlier study on the online learning environment found that effort regulation strategy was positively correlated with academic performance (Broadbent and Poon, 2015). In an online learning environment, one of the reasons for learners' success is their persistence in the face of distractions or obstacles when watching videos or performing boring tasks (Lee et al., 2019). Secondly, the simple and complex cognitive strategies combine to cognitive strategy factor. As suggested by the slow progress of research on interventions for the complex cognitive strategy, it is complex. Additionally, it is difficult to distinguish the complex strategy from other cognitive strategies, particularly in large-scale samples. Consistent with prior research (Kocdar et al., 2018) on self-regulated learning in open and distributed distance education, self-regulated learning strategies include self-learning, goal setting, help-seeking, environment management, and effort regulation strategies.

The COVID-19 pandemic has resulted in schools shut all across the world. Education has changed dramatically, with the distinctive rise of online learning, whereby teaching is undertaken remotely and on digital platforms. The integration of information technology in education will be further accelerated and that online education will eventually become an integral component of school education. The lack of structured in-class learning settings may have required more self-regulation and self-motivation to learn with less support. Given these challenges for educators, students, and parents, this study investigated the reliability and validity of self-regulated learning strategy measurement. It contributed to monitor students' use of learning strategies during online learning. Parents and teachers can provide timely guidance to ensure the effectiveness of learning.

There are some limitations in this study. First, the reliability and validity of the questionnaire needs to be further tested in different language and cultures. Second, the cross-sectional research design would not draw causality conclusions. Third, all the variables are self-reporting. The participants may overestimate theirs usage of strategies and introduce bias.

## Conclusion

With the popularization and application of online learning, the use of a localized perspective to understand the phenomenon of online learning is of great significance to online education research. This study examined the reliability and validity of Chinese version of the MSLQ-B-DL. For Chinese adult distance learners, this revised 20-item Chinese version of the questionnaire can effectively assess the quality of their learning strategies and lay the foundation for predicting their learning performance. Future research can be carried out in social science courses and STEM courses, respectively, to further verify the reliability and validity of the questionnaire.

## Data Availability Statement

The raw data supporting the conclusions of this article will be made available by the authors, without undue reservation.

## Ethics Statement

The studies involving human participants were reviewed and approved by Ethics Committee of Beijing Open University. The patients/participants provided their written informed consent to participate in this study.

## Author Contributions

Both authors listed have made a substantial, direct and intellectual contribution to the work, and approved it for publication.

## Conflict of Interest

The authors declare that the research was conducted in the absence of any commercial or financial relationships that could be construed as a potential conflict of interest.
