# Supplementary material for: Psychometric Properties of the MSLQ-B for Adult Distance Education in China
Source: Front Psychol. 2021 Feb 23;12:620564. doi: 10.3389/fpsyg.2021.620564 (PMC7940182; doi:10.3389/fpsyg.2021.620564)
Supplement: Supplementary file 1 [file Table_1.docx]

Items of Motivated Strategies for Learning Questionnaire part-B for distance learning (English)

| Items |  |
| --- | --- |
| 70 | I make sure that I keep up with the weekly readings and assignments for this course. |
| 73 | I attend this course regularly. |
| 74 | Even when course materials are dull and uninteresting, I manage to keep working until I finish. |
| 59 | I memorize key words to remind me of important concepts in this course. |
| 61 | I try to think through a topic and decide what I am supposed to learn from it rather than just reading it over when studying for this course. |
| 64 | When reading for this course, I try to relate the material to what I already know. |
| 76 | When studying for this course I try to determine which concepts I don't understand well. |
| 81 | I try to apply ideas from course readings in other course activities such as lecture and discussion. |
| 63 | When I study for this course, I go over my course notes and make an outline of important concepts. |
| 67 | When I study for this course, I write brief summaries of the main ideas from the readings and my course notes. |
| 72 | I make lists of important items for this course and memorize the lists. |
| 45 | I try to work with other students from this course to complete the course assignments. |
| 50 | When studying for this course, I often set aside time to discuss course material with a group of students from the course. |
| 58 | I ask the instructor to clarify concepts I don't understand well. |
| 75 | I try to identify students in this course whom I can ask for help if necessary. |
| 36 | When reading for this course, I make up questions to help focus my reading. |
| 38 | I often find myself questioning things I hear or read in this course to decide if I find them convincing. |
| 32 | When I study the readings for this course, I outline the material to help me organize my thoughts. |
| 60 | When course work is difficult, I either give up or only study the easy parts. (REVERSED) |
| 80 | I rarely find time to review my notes or readings before an exam. (REVERSED) |

Items of Motivated Strategies for Learning Questionnaire part-B for distance learning (Chinese)

| Items |  |
| --- | --- |
| 70 | 我确定跟上了这门课每周的阅读和作业节奏。 |
| 73 | 我有规律地按时学习这门课。 |
| 74 | 即使课程资源枯燥，我也会努力坚持直到完成学习任务。 |
| 59 | 我会熟记这门课程中的关键概念便于理解很多重要内容。 |
| 61 | 我会预先思考自己希望从这门课程中学到什么，而不是等到学习的时候才它从头到尾读一遍。 |
| 64 | 在阅读学材时，我试着把这些新知识与我记忆中的旧经验联系起来。 |
| 76 | 在学习时，我会找出那些还不太理解的概念知识点。 |
| 81 | 我会把从课程中获得的知识灵活地运用到活动或作业（如报告和讨论）中。 |
| 63 | 在学习时，我会复习我的笔记，并把重要的概念列成一个提纲。 |
| 67 | 在学习时，我会简要总结学材和笔记的中心思想和主要观点。 |
| 72 | 我会把这门课的重要内容整理出来，并把它们背下来。 |
| 45 | 我会和同学一起来完成这门课的作业。 |
| 50 | 在学习时，我经常和同学们相互探讨。 |
| 58 | 当遇到不太明白的知识点，我会请教辅导老师进一步讲解。 |
| 75 | 如果需要的话，我会主动去结识在这门课程中可以帮助我的同学。 |
| 36 | 在阅读学材时，我会给自己提一些问题来帮助自己集中注意力。 |
| 38 | 我会经常对课程中听到或读到的观点提出质疑，通过批判性思考以确定这些观点的合理性。 |
| 32 | 学习时，我会列出目录提纲以帮助自己理清思路。 |
| 60 | 当遇到困难的题目或难以自学的知识时，我要么选择放弃，要么只学习容易的部分。（反向） |
| 80 | 我很少在考试前复习笔记或书本。（反向） |
